# Enhancing the therapeutic activity of hyperimmune IgG against chikungunya virus using FcγRIIIa affinity chromatography

**DOI:** 10.3389/fimmu.2023.1153108

**Published:** 2023-05-12

**Authors:** Julie M. Fox, Vicky Roy, Bronwyn M. Gunn, Glen R. Bolton, Daved H. Fremont, Galit Alter, Michael S. Diamond, Austin W. Boesch

**Affiliations:** ^1^ Department of Medicine, Washington University in St. Louis, St. Louis, MO, United States; ^2^ Laboratory of Viral Diseases, National Institute of Allergy and Infectious Diseases, National Institutes of Health, Bethesda, MD, United States; ^3^ Ragon Institute of Massachusetts General Hospital (MGH), Massachusetts Institute of Technology (MIT), and Harvard University, Cambridge, MA, United States; ^4^ Zepteon, Inc., Boston, MA, United States; ^5^ Department of Pathology and Immunology, Washington University in St. Louis, St. Louis, MO, United States; ^6^ Department of Molecular Microbiology, Washington University in St. Louis, St. Louis, MO, United States; ^7^ Moderna, Inc., Cambridge, MA, United States

**Keywords:** glycoform, antibody effector function, afucosylated, FcγRIIIa, chromatography, hyperimmune IgG, chikungunya virus

## Abstract

**Introduction:**

Chikungunya virus (CHIKV) is a re-emerging mosquito transmitted alphavirus of global concern. Neutralizing antibodies and antibody Fc-effector functions have been shown to reduce CHIKV disease and infection in animals. However, the ability to improve the therapeutic activity of CHIKV-specific polyclonal IgG by enhancing Fc-effector functions through modulation of IgG subclass and glycoforms remains unknown. Here, we evaluated the protective efficacy of CHIKV-immune IgG enriched for binding to Fc-gamma receptor IIIa (FcγRIIIa) to select for IgG with enhanced Fc effector functions.

**Methods:**

Total IgG was isolated from CHIKV-immune convalescent donors with and without additional purification by FcγRIIIa affinity chromatography. The enriched IgG was characterized in biophysical and biological assays and assessed for therapeutic efficacy during CHIKV infection in mice.

**Results:**

FcγRIIIa-column purification enriched for afucosylated IgG glycoforms. In vitro characterization showed the enriched CHIKV-immune IgG had enhanced human FcγRIIIa and mouse FcγRIV affinity and FcγR-mediated effector function without reducing virus neutralization in cellular assays. When administered as post-exposure therapy in mice, CHIKV-immune IgG enriched in afucosylated glycoforms promoted reduction in viral load.

**Discussion:**

Our study provides evidence that, in mice, increasing Fc engagement of FcγRs on effector cells, by leveraging FcγRIIIa-affinity chromatography, enhanced the antiviral activity of CHIKV-immune IgG and reveals a path to produce more effective therapeutics against these and potentially other emerging viruses.

## Introduction

Chikungunya virus (CHIKV) is an enveloped, mosquito-borne virus in the *Alphavirus* genus and *Togaviridae* family. CHIKV was first isolated in 1952 in Tanzania and, until recently, primarily caused outbreaks in Africa and Asia. In 2013, CHIKV emerged in the Caribbean and spread into Central and South America. By 2017, over 2.5 million suspected cases were reported throughout the Americas (1–3). CHIKV infection can result in rash, fever, myalgia, and debilitating polyarthritis and polyarthralgia. For most patients, symptoms resolve after several weeks. However, a subset (~10 to 30%) of patients develop persistent joint pain that can last for multiple years following infection (4, 5). While there are no therapies licensed to treat CHIKV disease, numerous studies have shown significant protection with antibody treatment in animal models.

Hyperimmune immunoglobulin therapy involves the administration of purified and concentrated immunoglobulins from convalescent donors that have high antibody titers against the pathogen of interest, with the main component being IgG (6, 7). Such therapies are approved against several viruses including cytomegalovirus, hepatitis A, hepatitis B, rabies, and varicella zoster viruses, but their development against emerging viruses such as influenza, Ebola, Zika, and SARS-CoV-2 has been challenged by lack of efficacy or the potential for antibody-dependent enhancement (ADE) of infection (8–12). While hyperimmune IgG binds specific viral antigen resulting in direct neutralization, *in vivo* studies have determined that interaction of the IgG Fc region with FcγRs on immune effector cells can enhance therapeutic protection during Ebola, influenza, SARS-CoV-2 and CHIKV infection (13–20). The interaction between IgG-Fc and FcγRs can be modulated based on the IgG subclass and Fc glycoforms, ultimately impacting the antibodies functionality and therapeutic potential (21–24).

The use of Fc-engineered antibodies with enhanced FcγR binding has improved the therapeutic efficacy *in vivo* against Ebola, influenza, SARS-CoV-2, and respiratory syncytial viruses (16, 25–30). However, antibodies with enhanced FcγR engagement activity were ineffective against HIV and shown to augment disease severity in Dengue *in vivo* due to ADE of infection highlighting the importance of carefully optimizing the IgG-Fc-FcγR interactions depending on the role antibodies play in disease pathogenesis (31, 32). Antibodies against CHIKV can interact and neutralize free virus in solution and bind the viral glycoproteins present on the surface of infected cells and prevent virion morphogenesis and budding. The antibody interactions with infected cells can also engage complement and FcγRs resulting in immune-mediated clearance mechanisms, such as complement activation, antibody-dependent cellular phagocytosis (ADCP), antibody dependent cellular cytotoxicity (ADCC), and antibody-dependent cell-mediated viral inhibition (ADCVI).

CHIKV-specific monoclonal antibodies and hyperimmune IgG have been shown to provide protection in immunocompetent and immunocompromised animal models through multiple mechanisms including neutralization and Fc-effector functions (13, 33–36). However, the ability to improve therapeutic activity of CHIKV-specific antibodies through enhanced FcγR engagement remains unknown. A previous report described a method of separating fucosylated and afucosylated antibodies using an affinity resin containing immobilized FcγRIII (CD16) receptors (37). This method was used successfully to enrich for afucosylated IgG and generated IgG glycoform and subclass mixtures with optimized effector functions relative to the starting serum IgG (38–40).

Here, using a murine model of CHIKV-induced arthritis, we evaluated the therapeutic activity of CHIKV-immune IgG enriched for glycoforms with enhanced binding to Fc receptors by utilizing FcγRIIIa-based affinity chromatography. Additional biophysical and biological assays were performed to evaluate the differences in IgG glycoforms, mouse and human FcγR binding, IgG subclass distribution, virus neutralization, and monocyte/neutrophil phagocytosis. The FcγRIIIa-chromatography enriched IgG boosted the clearance of infectious virus and viral RNA compared to non-enriched IgG. Glycan and IgG subclass analysis revealed that CHIKV-immune IgG that bound human FcγRIIIa were enriched for afucosylated antibodies, which correlated with enhanced neutrophil phagocytic activity using beads coated with CHIKV structural proteins. Overall, these studies demonstrate the utility of FcγRIIIa-affinity chromatography to alter the Fc-mediated effector functions of hyperimmune IgG and optimize the therapeutic activity of antibodies against CHIKV.

## Methods

### Cells and viruses

C6/36 and Vero cells were purchased from the American Type Culture Collection (ATCC) and cultured as described previously (34). The CHIKV La Reunion OPY1 strain was generated from a cDNA clone and passaged on C3/36 cells, as previously described (13, 41).

### Mouse studies

Experiments were approved by the Institutional Animal Care and Use Committee at the Washington University School of Medicine (Assurance Number: A3381-01) in accordance with the recommendations in the Guide for the Care and Use of Laboratory Animals of the National Institutes of Health. All injections with virus were administered under anesthesia with ketamine hydrochloride (80 mg/kg) and xylazine (15 mg/kg).

Four-week-old female and male wild type (WT) C57BL/6J mice were purchased from Jackson Laboratories. Mice were infected with 10^3^ FFU of CHIKV diluted in Hank’s Balanced Salt Solution (HBSS) supplemented 1% heat-inactivated (HI)-FBS *via* subcutaneous inoculation in the left footpad. One day after infection, mice were administered polyclonal IgG or PBS by intraperitoneal injection. Swelling of the ipsilateral foot was measured (width x height) using digital calipers. Tissues were collected following extensive PBS perfusion at 5 days post-infection (dpi) and titrated by focus-forming assay on Vero cells using a murine anti-CHIKV mAb, CHK-11, as the detection antibody or by RT-qPCR and normalized to tissue weight, as previously described (34, 42). For RT-qPCR analysis, focus forming unit (FFU) equivalent was determined using a standard curve with RNA isolated from viral stocks.

### Enrichment of afucosylated human anti-CHIKV IgG

Human anti-CHIKV polyclonal IgG was purified by protein G from donor plasma collected in the convalescent phase of CHIKV infection as part of an IRB-approved study (Washington University Arthritis and Rheumatology-Tissue Procurement Facility IRB-approved protocol (43)). A total of 121 vials of frozen CHIKV-immune plasma (160 mL) corresponding to 10 donors was thawed, pooled, centrifuged (2,200 x g, 10 minutes), and clarified through a 0.2 μm polyethersulfone filter. A Protein G resin (GenScript L00209, 10 ml) was packed in a 1.6 cm x 5 cm bed, washed with 50 mM glycine pH 3.5 and then equilibrated in PBS. Plasma was loaded onto the column (monitored at 280 nm) at 3 mL/min, then washed extensively with PBS. The resin was washed with five column volumes of PBS with 0.5 M NaCl, five column volumes of PBS, and protein was eluted with 50 mM glycine pH 3.1 and neutralized with 1 M 2-(N-Morpholino)-Ethanesulfonic acid (MES) pH 6.3 (final ratio of 4:1 elute to MES). The neutralized eluate then was passed through a 0.2 μm PES filter and stored at 4°C.

The purified eluate then was diluted ten-fold in PBS to a final concentration of 2 mg/mL. A 2-mL Glycap 3A (human FcγRIIIa) affinity column (0.5 cm x 10 cm; Zepteon) was equilibrated in PBS. An aliquot of the diluted IgG was loaded onto the column (monitored at 280 nm) at 1 mL/min. The column was washed sequentially with PBS, ten column volumes of PBS with 0.5 M NaCl, ten column volumes of PBS, and protein was eluted with 50 mM glycine pH 3.5 and neutralized with 1 M MES pH 6.3 (final ratio of ~4.5:1 elute to MES). The column was then re-equilibrated in PBS, and the purification was repeated with fresh aliquots of the diluted IgG. Unbound IgG was collected from each run, and re-run over column to purify any breakthrough product. Neutralized eluates were pooled from separate column runs and clarified through a 0.2 µm PES filter and stored at 4°C. Pooled eluate was concentrated on a 30 kDa Amicon Ultra15 (Millipore UFC903024). Concentrated eluate and the Glycap 3A column Load material was dialyzed in PBS in a 10 kDa Slide-a-Lyzer (Thermo 66810).

### Glycan profiling using capillary electrophoresis

Capillary electrophoresis was used to quantify the relative abundance of antibody glycan structures as previously described (44). Briefly, hyperimmune IgG was bound to protein G immobilized on magnetic beads (New England Biolabs) and then incubated with IdeZ protease (New England Biolabs) to release the IgG-Fab portion. The beads still bound with IgG-Fc were washed, and N-glycans were released from the IgG-Fc and labeled with 8-aminopyrene-1,3,6-trisulfonic acid (APTS) using the GlycanAssure APTS Kit (Thermo Fisher Scientific), as described in the manufacturer’s protocol. Labeled glycans were loaded and analyzed by the 3500 Genetic Analyzer (Thermo Fisher Scientific). Peaks for sialylated, fucosylated, galactosylated, and bisected (GlcNAc) structures were identified. The relative amount of each glycan structure was determined by calculating the area under the curve (AUC), which was normalized to the loaded APTS dye, for each peak then divided by the total area of all peaks. The percent enrichment was determined using the formula: [-(relative abundance of CHIKV load IgG – relative abundance of CHIKV eluate IgG)/relative abundance in the CHIKV load IgG]*100.

### FcγR ELISA

Human FcγRIIIa, mouse FcγRIII, or mouse FcγRIV recombinant protein (R&D Systems) were diluted in a sodium bicarbonate buffer (pH 9.3) and adsorbed overnight at 4°C on a Maxisorp immunocapture ELISA plate. Wells were washed with PBS with 0.05% Tween 20 then incubated in blocking buffer (PBS + 2% BSA (Sigma)) for 1 h at 37°C. Hyperimmune IgG samples were serially diluted in blocking buffer and added to wells for 2 h at room temperature. The wells were washed then incubated with an HRP-conjugated goat anti-human kappa antibody (Southern Biotech) for 1 h at room temperature. Following washes, TMB one-step substrate system (Dako) was used to develop the assay. The reaction was stopped with 1 M H_2_SO_4_ and absorbance was collected at 450 nm. Mouse and human mAbs were included as positive controls for each protein. The endpoint dilution, which is the lowest antibody concentration to provide a positive signal (defined by the OD value for the no antibody control plus two times the standard deviation), was determined using non-linear regression.

### Neutralization assay

Focus reduction neutralization tests (FRNT) were performed as previously described using CHK-11 as the detection antibody (42). IC50 values were determined using non-linear regression constraining the top and bottom to 100 and 0, respectively.

### Human IgG ELISA

Unlabeled goat anti-human kappa chain antibody was adsorbed on a Maxisorp immunocapture ELISA plate in a sodium bicarbonate buffer pH 9.3 overnight at 4°C. Wells were washed with PBS with 0.05% Tween 20 and incubated with blocking buffer for 1 h at 37°C. Hyperimmune IgG samples were diluted in blocking buffer and added to wells for 1 h at room temperature. Plates were washed then incubated with biotin conjugated mouse anti-human IgG1, mouse anti-human IgG2, mouse anti-human IgG3, mouse anti-human IgG4, or goat anti-human IgG Fc- multiple species cross-adsorbed antibody for 1 h at room temperature. Wells were washed and incubated with HRP-conjugated streptavidin (Vector) for 1 h at room temperature. Following washing, plates were developed as described above. Similar binding patterns were observed after coating with unlabeled goat anti-human lambda antibody (data not shown). The endpoint dilution was determined as described above.

### CHIKV capture ELISA

CHK-152 was diluted in a sodium bicarbonate buffer (pH 9.3) and adsorbed overnight at 4°C on a Maxisorp immunocapture ELISA plate. Wells were washed with PBS with 0.05% Tween 20 and incubated with blocking buffer for 1 h at 37°C. CHIKV 181-25 was captured for 1 h at room temperature. Hyperimmune IgG samples were serially diluted in blocking buffer, added to wells, and incubated for 1 h at room temperature. Plates were washed and then incubated with biotin-conjugated mouse anti-human IgG1 (Southern Biotech clone 4E3), mouse anti-human IgG2 (Southern Biotech clone HP6002), mouse anti-human IgG3 (Southern Biotech clone HP6050), mouse anti-human IgG4 (Southern Biotech clone HP6023), or goat anti-human IgG Fc-multiple species cross-adsorbed (Southern Biotech) antibody for 1 h at room temperature. Wells were washed and incubated with HRP-conjugated streptavidin at room temperature for 1 h. Following washing, the assay was developed as described for above. The endpoint dilution was determined as described above.

### Preparation of recombinant CHIKV p62-E1

CHIKV (strain La Reunion) p62-E1 (E3-E2-E1: residues S1-R64 of E3, S1-E161 of E2, and Y1-Q411 of E1 including a (GGGS)_4_ polylinker between E2 and E1) was prepared as previously described (13).

### Antibody-dependent cellular phagocytosis

The ADCP assay was adapted from a previously described method (45). Briefly, recombinant biotinylated CHIKV p62-E1 conjugated to streptavidin-coated Alexa Flour 488 beads (Invitrogen) for 2 h at 37°C and washed two times in 0.1% BSA in PBS. CHIKV p62-E1-coated beads were incubated with polyclonal antibodies (5-fold dilutions) in culture medium for 2 h at 37°C. The human THP-1 monocytic cell line (hADCP) or monocytes harvested and purified from the bone marrow of C57BL/6 mice using a CD14^+^ enrichment kit (Stem Cell Technologies) (mADCP) were added at a concentration of 2.5 × 10^4^ cells/well and incubated for 4 h at 37°C (mADCP) or overnight (hADCP) in low adherence 96-well plates (mADCP) or regular 96-well assay plates (hADCP). Following incubation, mouse monocytes (mADCP) were incubated with the following antibodies: Ly6G BV421 (BioLegend clone 1A8), CD11c AlexaFluor700 (BioLegend clone N418), and CD11b APC (BioLegend clone M1/70). After fixing the cells with 4% PFA, they were analyzed by flow cytometry (BD LSRII). The data was collected using Diva and analyzed using FlowJo software. The phagocytic score was calculated as follows: (% of Alexa Fluor 488 cells) x (Alexa Fluor 488 geometric mean fluorescent intensity (MFI) of Alexa Fluor 488 cells)/10,000.

### Antibody-dependent complement deposition

The ADCD assay was adapted from a previously described method (46). Briefly, biotinylated antigen was bound to 1 μm fluorescent Neutravidin beads (Invitrogen). Immune complexes were formed by incubating 10 μL of antigen-bound beads with 10 μL of diluted hyperimmune IgG for 2 hours at 37˚C. The immune complexes were spun down and washed with PBS. Lyophilized complement from guinea pig (Cedarlane) was resuspended in cold water, diluted in Gelatin Veronal Buffer (Boston BioProducts) and added to the immune complexes. The samples were incubated for 50 minutes at 37˚C and the reaction was quenched by washing the plates twice with 15mM EDTA in PBS. For complement deposition detection, samples were incubated for 15 min with Fluorescein-conjugated goat anti-guinea pig complement C3 (MP Biomedicals) in the dark and the samples were analyzed by flow cytometry (BD LSRII). The level of complement deposition was calculated as the ratio relative to naïve levels, which were set to 1.

### Antibody-dependent neutrophil phagocytosis

The ADNP assay was adapted from a previous publication (47). Briefly, recombinant, biotinylated CHIKV p62-E1 protein was conjugated to streptavidin-coated Alexa Fluor 488 beads (Invitrogen) for 2 h at 37°C. After washing two times in 0.1% BSA in PBS, CHIKV antigen–coated beads were incubated with hyperimmune IgG (5-fold dilutions) in cell culture medium for 2 h at 37°C to form immune complexes. Bone marrow cells were harvested from C57BL/6 mice (mADNP) or primary neutrophils were isolated from fresh ACD blood (hADNP). Cells were washed with PBS, and 5.0 × 10^4^ cells per well were added to the immune complexes and incubated for 1 h at 37°C. The following antibodies were used to stain the cells: mADNP: CD11c APC/Cy7 (clone N418; BioLegend), CD11b APC (clone M1/70; BioLegend), Ly-6C BV605 (clone HK1.4; BioLegend), Ly6G Pacific Blue (clone 1A8; BioLegend), and CD3 PE/Cy7 (clone 17A2; BioLegend) or hADNP: CD66b Pacific Blue (clone G10F5; BioLegend). Cells were fixed with 4% PFA and analyzed on a BD LSRII flow cytometer. Neutrophils were defined as CD3^−^ and CD11c^−^ cells that were Ly6C^−^, CD11b^+^, and Ly6G^+^ for mouse cells or CD66b^+^ for human cells. The phagocytic score was calculated as follows: (percentage of Alexa Fluor 488^+^ cells) × (Alexa Fluor 488 geometric mean fluorescent intensity of Alexa Fluor 488^+^ cells)/10,000.

### Statistical analysis

Statistical significance was determined using GraphPad Prism version 7.0 and 9.0 (La Jolla, CA) and assigned when the *P* value < 0.05. The statistical test used for each data set is specified in figure legend. Nonlinear regression was performed using GraphPad Prism version 9.0. An unpaired t-test was used to determine significance between the endpoint dilution values for each group in the FcγR, human IgG, and CHIKV capture ELISAs. An unpaired t-test was used to determine significance between the IC50 values. A two-way ANOVA with a Tukey’s post-test (between all groups) was used to determine significance for foot swelling analysis. A one-way ANOVA with a Tukey’s post-test was used to determined significance for most of the virological analysis. A Kruskal-Wallis test with a Dunn’s post-test was used to determine differences in the infectious virus burden in the contralateral ankle because values were at the limit of detection. AUC analysis was performed for the ADCD, ADCP, and ADNP assays and a one-way ANOVA with a Tukey’s post-test (between all groups) was used to determine significant differences.

### Materials availability

Requests for reagents, including antibodies, proteins, viruses, and primer-probe sets, and resources should be directed to the corresponding authors. The reagents will be made available on request after completion of a Materials Transfer Agreement (MTA).

## Results

### Anti-CHIKV antibodies enriched by hFcγRIIIa-affinity chromatography promote reductions in viral burden

**Figure 1:**
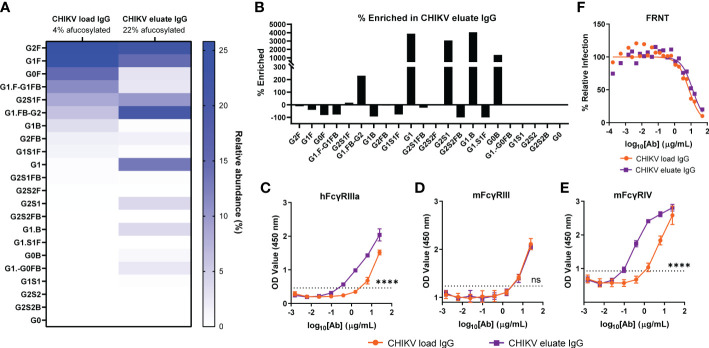
Enrichment of afucosylated anti-CHIKV antibodies by binding immobilized human FcγRIIIa. Total IgG was purified from CHIKV-immune human plasma (CHIKV load IgG) and further fractionated for binding to a human FcγRIIIa resin (CHIKV eluate IgG). **(A)** The relative abundance of the N-linked glycan patterns isolated from the CHIKV eluate IgG and CHIKV load IgG was determined by capillary electrophoresis. Nomenclature of glycans: G0, G1, G2, contain 0, 1, 2 galactose residues, respectively; F, fucose; S, sialic acid; B, bisecting N-acetylglucosamine. **(B)** The percent enriched of specific glycan patterns in CHIKV eluate IgG compared to CHIKV load IgG. **(C-E)** Binding of CHIKV eluate IgG and CHIKV load IgG to **(C)** human (h) FcγRIIIa, **(D)** mouse (m) FcγRIII, or **(E)** mFcγRIV by ELISA The dotted line indicates the background threshold for a positive signal to determine the endpoint dilution. Statistical significance was determined between the endpoint dilution value for each group (2 independent experiments, ****P < 0.0001; ns, not significant; **(C–E)**: unpaired t-test). **(F)** CHIKV eluate IgG and CHIKV load IgG were assayed for neutralizing activity (3 independent experiments).

Glycosylation of the antibody heavy chain modulates FcγR interactions and effector functions (48, 49). For humans, antibodies lacking a core fucose on their N-acetylglucosamine moieties exhibit increased ADCC through improved binding to human FcγRIIIa for NK cells, ADCC/ADCP *via* FcγRIIIa for monocytes and macrophages, and ADCP by FcγRIIIb for neutrophils (21, 22, 50). For mice, the FcγRIIIa ortholog, FcγRIV, also preferentially binds afucosylated IgG but is restricted to mouse monocytes/macrophages and neutrophils, each of which is capable of eliciting ADCC and ADCP mediated by human IgG (13, 51–55). Since anti-CHIKV mAbs required effector functions for optimal reduction of viral burden, we evaluated whether afucosylated anti-CHIKV antibodies could augment viral clearance (13). We purified total IgG from pooled CHIKV immune plasma (CHIKV load IgG) and secondarily fractionated it using an immobilized human FcγRIIIa resin (CHIKV eluate IgG) (37, 43). The glycan composition of the load and eluate IgG was determined by capillary electrophoresis (**Figure 1A**). Whereas the load material had a 4% fraction of afucosylated Fc, the eluate was enriched with a 22% fraction of afucosylated Fc (**Figure 1A**). This is a 449% enrichment of afucosylated Fc in the CHIKV eluate IgG group. Fractionation with immobilized human FcγRIIIa, enriched for specific glycoforms including G1, G2S1, G1.B, and G0B (**Figure 1B**). As expected, the afucosylated enriched CHIKV eluate IgG showed enhanced binding to human FcγRIIIa compared to the CHIKV load IgG (**Figure 1C**). The CHIKV IgG in the eluate also bound more avidly to mouse FcγRIV but not mouse FcγRIII (**Figures 1D, E**) consistent with previous observations for afucosylated IgG (55). Notwithstanding these glycosylation differences, the load and eluate antibodies neutralized CHIKV similarly (IC50 values (95% confidence interval): CHIKV load IgG 7.7 µg/mL (6.1 - 9.9) and CHIKV eluate IgG 12.9 µg/mL (9.7 - 18.0), *P > 0.05*) in cell culture (**Figure 1F**).

**Figure 2:**
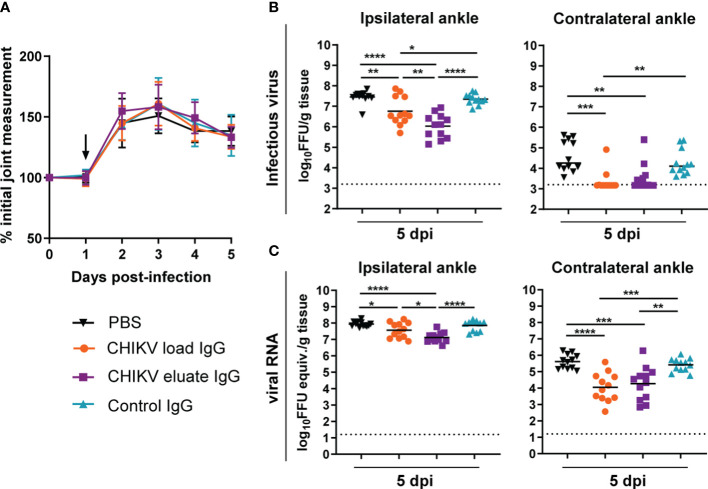
Anti-CHIKV antibodies enriched for binding to human FcγRIIIa promote reduction in viral load. Four-week-old male and female WT C57BL/6 mice were inoculated with 10^3^ FFU of CHIKV and, at 1 dpi, administered 100 µg of CHIKV eluate IgG, CHIKV load IgG, non-immune IgG purified from healthy controls (control IgG), or PBS. **(A)** Foot swelling was measured prior to infection and for the following five days. The arrow indicates the day the IgG or PBS was administered. **(B, C)** Ipsilateral and contralateral ankles were collected at 5 dpi, and **(B)** infectious virus and **(C)** viral RNA load were determined by FFA or qRT-PCR, respectively. **(A–C)**: n = 12, 3 independent experiments, *P < 0.05; **P < 0.01; ***P < 0.001; ****P < 0.0001; **(B)**, *left*, and **(C)**: one way ANOVA with a Tukey’s post-test; **(B)**, *right*: Kruskal-Wallis with a Dunn’s post-test). The dotted line indicates the limit of detection for the assay.

We compared the protective activity of the load and afucosylated enriched eluate anti-CHIKV IgG in the mouse model of CHIKV-induced musculoskeletal disease and arthritis. Since the overall neutralization capability was reduced relative to studies with potently neutralizing anti-CHIKV mAbs, we administered the polyclonal anti-CHIKV IgG at 1 dpi (13). Although administration of the load and eluate CHIKV IgG did not change foot swelling (**Figure 2A**), both reduced the levels of infectious virus and viral RNA in the ipsilateral and contralateral ankle at 5 dpi compared to the PBS or non-immune control IgG groups (**Figures 2B, C**). However, the inhibitory effects on infectious viral yield (8-fold, *P* < 0.01) or viral RNA levels were greater (3-fold, *P* < 0.05) with the afucosylated enriched eluate than with the load CHIKV IgG at the site of inoculation (*e.g.*, ipsilateral ankle). One possible explanation for the greater therapeutic activity of the eluate CHIKV IgG is differences in the IgG subclass composition.

**Figure 3:**
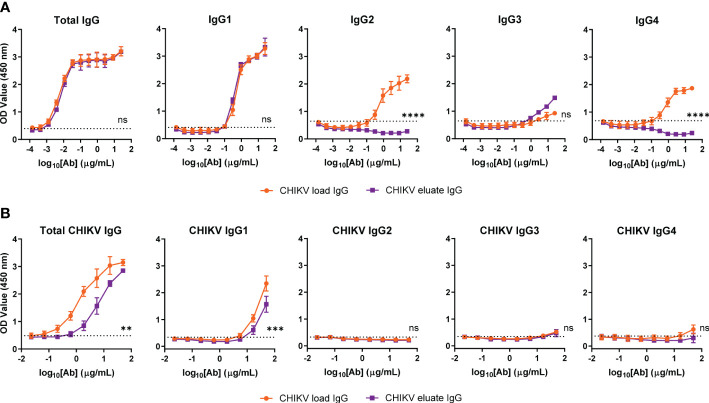
Similar CHIKV-specific IgG subclasses present in CHIKV eluate and load IgG. Levels of **(A)** total or **(B)** CHIKV-specific IgG subclasses in CHIKV eluate IgG and CHIKV load IgG as determined by ELISA. The dotted line indicates the background threshold for a positive signal to determine the endpoint dilution. Statistical significance was determined between the endpoint dilution value for each group (2 independent experiments, **P < 0.01, ***P < 0.001; ****P < 0.0001; ns, not significant; unpaired t-test).

To address this issue, we performed subclass analysis of the total IgG and CHIKV-specific IgG from the load and eluate groups. For total IgG, the human FcγRIIIa resin left IgG1 unchanged, modestly enriched IgG3, and eliminated human IgG2 and IgG4, as expected based on previous studies (38) (**Figure 3A**); however, the majority of the CHIKV specific IgG in both the load and eluate groups was human IgG1 (**Figure 3B**). Notwithstanding this result, the CHIKV eluate IgG had significantly reduced levels of CHIKV-specific IgG and IgG1 compared to the CHIKV load IgG. Since there was similar virus neutralization between the CHIKV eluate and load IgG, these results suggest that the antibodies enriched using the human FcγRIIIa resin were more functional which resulted in greater virus reduction *in vivo*.

### Afucosylated enriched anti-CHIKV antibodies enhance neutrophil phagocytosis

**Figure 4:**
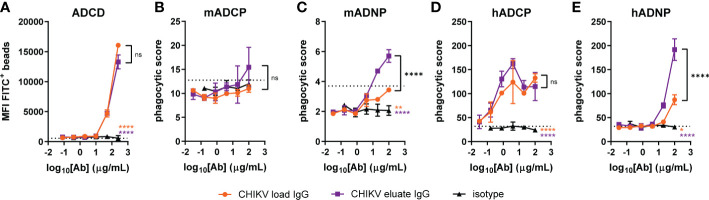
Increased neutrophil phagocytosis with anti-CHIKV antibodies enriched using human FcγRIIIa-chromatography. CHIKV load IgG, CHIKV eluate IgG, or an isotype control were evaluated for **(A)** complement deposition (C3b) on CHIKV p62-E1-coated beads (ADCD: antibody-dependent complement deposition), **(B, C)** mouse (m) or **(D, E)** human (h) **(B, D)** monocyte or **(C, E)** neutrophil-directed phagocytosis of CHIKV p62-E1 coated-beads (ADCP, antibody-dependent cellular phagocytosis; ADNP, antibody-dependent neutrophil phagocytosis). The dotted line indicates the no antibody control. Statistical significance was determined between the AUC analysis for each group (2 independent experiments, *P < 0.05; **P < 0.01; ****P < 0.0001; ns, not significant; one-way ANOVA with a Tukey’s post-test). The black stars indicate significance between CHIKV load IgG and CHIKV eluate IgG, the orange stars indicate significance between CHIKV load IgG and isotype, and the purple stars indicate significance between CHIKV eluate IgG and isotype.

To gain insight into the basis for why the CHIKV eluate IgG showed greater infectious virus/viral RNA reduction than the CHIKV load IgG *in vivo*, we performed an *in vitro* analysis of Fc effector functions using complement components and mouse immune cells with CHIKV p62-E1 protein coated beads. CHIKV eluate IgG and CHIKV load IgG significantly increased complement deposition, but no difference was observed between the two CHIKV-immune IgG preparations (**Figure 4A**). Surprisingly, neither CHIKV-immune IgG preparation enhanced phagocytosis of CHIKV antigen-coated beads by primary mouse monocytes compared to the isotype control (**Figure 4B**). The major effector function difference between CHIKV eluate and load IgG was phagocytosis by mouse neutrophils. Although CHIKV load IgG increased phagocytosis compared to the isotype control, the phagocytic score was still below background levels (**Figure 4C**). In contrast, incubation of the antigen-coated beads with CHIKV eluate IgG increased neutrophil phagocytosis over background levels as well as the isotype control and CHIKV load IgG (**Figure 4C**).

We next assessed effector function activity using human immune cells. CHIKV eluate IgG and CHIKV load IgG increased monocyte phagocytosis of antigen-coated beads compared to the isotype control; however, CHIKV eluate IgG did not further enhance monocyte phagocytosis compared to CHIKV load IgG (**Figure 4D**). In agreement with the assays using mouse immune cells, CHIKV eluate IgG augmented human neutrophil phagocytosis compared to the CHIKV load IgG (**Figure 4E**). These results suggest that antibodies enriched by human FcγRIIIa-chromatography promoted clearance of viral RNA in mice, possibly through an enhanced interaction with murine neutrophils that express FcγRIV. A similar mechanism might occur in humans *via* FcγRIIIb, although additional cell types, such as monocytes, could aid in viral clearance (22).

## Discussion

Although prior studies have established the importance of Fc-FcγR interactions for efficacy of IgG therapy during CHIKV infection, the ability to improve the activity of antibody therapies by enhancing Fc-FcγR engagement *in vivo* have not been explored. In this study, we examined whether CHIKV-Ig enriched in afucosylated glycoforms with enhanced Fc effector functions could affect the outcome in a murine model of CHIKV infection. Polyclonal anti-CHIKV IgG enriched by human FcγRIIIa-chromatography promoted clearance of CHIKV infection in mice, and this correlated with enhanced phagocytosis of CHIKV antigen-coated beads by murine and human neutrophils. Collectively, our results suggest that optimizing specific Fc-FcγR interactions can enhance clearance of CHIKV infection.

Glycosylation patterns on the IgG heavy chain can influence the affinity to specific FcγRs and result in altered effector functions. Afucosylated antibodies bind with higher affinity to human FcγRIIIa and mouse FcγRIV, which can result in increased ADCC by NK cells and ADCC and ADCP by monocytes and macrophages (21, 50, 56, 57). Our experiments showed that polyclonal CHIKV immune IgG that was enriched for binding to human FcγRIIIa enhanced the clearance of infectious virus and viral RNA in the ipsilateral ankle compared to the CHIKV IgG load material. Based on the Fc effector function analysis, viral clearance may have been mediated by enhanced neutrophil phagocytosis of CHIKV-infected cells. Interestingly, the CHIKV eluate and load IgG reduced infectious virus and viral RNA in the contralateral ankle to a similar degree. This may be related to the timing of antibody administration. At one day post-infection, high CHIKV titers are present in the ipsilateral ankle, whereas the infection is still being established in more distal tissues, such as the contralateral ankle (58). For this reason, viral neutralization may be a more dominant mechanism in distal tissues compared to antibody effector functions. Alternatively, the infiltrating immune cell population may be different between the tissues, which could impact clearance of infected cells and ultimately the viral RNA burden. Additional studies will be needed to define correlates of protection in distal tissues. Although we observed greater viral load reduction with these CHIKV IgG glycoforms in wild-type mice, since FcγR expression pattern on immune cells, and their affinity for particular IgG subclasses, varies between mouse and humans, it will be important to confirm this protective effect in transgenic mice expressing human FcγRs (59). However, these data do highlight the parallels in IgG binding between human FcγRIIIa and murine FcγRIV that may inform bridging studies between the two species.

The purification and use of total IgG, rather than isolation of CHIKV-specific IgG, more closely represents the hyperimmune immunoglobulin used clinically, however, this limits the interpretation of the results. FcγRIIIa purification selected for IgG1 and IgG3 subclasses with IgG2 and IgG4 being eliminated. However, the main IgG subclass observed in both the CHIKV eluate and load IgG was IgG1, so we cannot comment on the impact of IgG subclass on CHIKV protection. Furthermore, we did not determine the relative enrichment of afucosylated glycoforms on CHIKV-specific IgG though methods do exist (60). Since the CHIKV IgG eluate and load groups neutralized CHIKV similarly, it is possible that much of the CHIKV-specific IgG was enriched. Additional studies will be necessary to evaluate the glycoforms, subclasses, and activity of FcγRIIIa binding enriched CHIKV-specific IgG and how this compares to the load sample and the non-enriched flow-through fraction.

Antibodies can limit CHIKV infection and disease progression, particularly when delivered prior to or shortly after virus infection (33–35, 61). The contribution of antibody Fc effector functions to protection has been observed in other viral infections (15, 29, 62–64). Moreover, broadly neutralizing mAbs targeting HIV, Ebola, RSV, influenza, and SARS-CoV-2 with modifications that enhance Fc-FcγR affinity increased antibody efficacy (16, 25, 27, 30, 65). Consistent with these studies, anti-CHIKV IgG enriched in predominantly afucosylated IgG-Fc *via* FcγRIIIa affinity chromatography exhibited enhanced therapeutic activity. An additional application worth exploring is the ability to deplete immune activating IgG glycoforms and subclasses by FcγRIIIa affinity chromatography to generate ADE-attenuated therapeutics to combat viruses such as Dengue. In summary, our study demonstrates a proof-of-principle for a facile chromatography method to produce hyperimmune IgG therapeutics against emerging viruses with enhanced activity.

## Data availability statement

The original contributions presented in the study are included in the article. Further inquiries can be directed to the corresponding authors.

## Ethics statement

The animal study was reviewed and approved by Institutional Animal Care and Use Committee at the Washington University School of Medicine (Assurance Number: A3381-01).

## Author contributions

JF, VR, and BG performed experiments. JF, BG, GB, GA, MD, and AB designed the experiments and analyzed the data. DF, GB, and AB contributed key reagents and methodology. JF, MD, and AB wrote the first draft of the manuscript, and all authors provided editorial comments. All authors contributed to the article and approved the submitted version.
